# Numerical Study on Thermodynamic Behavior during Selective Laser Melting of 24CrNiMo Alloy Steel

**DOI:** 10.3390/ma13010045

**Published:** 2019-12-20

**Authors:** Xiangpeng Luo, Minghuang Zhao, Jiayi Li, Chenghong Duan

**Affiliations:** College of Mechanical and Electrical Engineering, Beijing University of Chemical Technology, North Third Ring Road 15, Chaoyang District, Beijing 100029, China; xpluo@mail.buct.edu.cn (X.L.); zhaomh26@163.com (M.Z.); 18811409192@163.com (J.L.)

**Keywords:** selective laser melting, finite element analysis, thermodynamic behavior, substrate preheating, scanning strategy, 24CrNiMo alloy steel

## Abstract

In this paper, a multi-layer and multi-track finite element model of 24CrNiMo alloy steel by selective laser melting (SLM) is established by using the ABAQUS software. The distribution and evolution of temperature field and stress field and the influence of process parameters on them are systematically studied. The results show that the peak temperature increases from 2153 °C to 3105 °C and the residual stress increases from 335 MPa to 364 MPa with increasing laser power from 200 W to 300 W; the peak temperature decreases from 2905 °C to 2405 °C and the residual stress increases from 327 MPa to 363 MPa with increasing scanning speed from 150 mm/s to 250 mm/s; the peak temperature increases from 2621 °C to 2914 °C and the residual stress decreases from 354 MPa to 300 MPa with increasing preheating temperature from 25 °C to 400 °C. Far away from scanning area, far away from starting point, and the adjacent areas with vertical scanning direction, resulting in a uniform temperature distribution, help to reduce the residual stress. Due to the remelting effect, the interlayer scanning angle changing helps to release the residual stress of the former layer causing a smaller residual stress after redistribution.

## 1. Introduction

As an academic term for 3D printing, rapid prototyping, and layer manufacturing, additive manufacturing (AM) combines materials processing and forming technology, computer-aided design, and so on. Based on the principle of discrete-stacking, this technology stacks metal materials or non-metal materials by layers to directly produce three-dimensional solid parts through software and control systems, by means of sintering, melting, spraying, photocuring, etc. Compared with the traditional subtractive manufacturing of cutting raw materials, AM is a manufacturing method that is able to process three-dimensional complex structural parts by adding materials point by point, line by line, and surface by surface [1,2,3]. Moreover, AM technology has the advantages of flexible processing, short production cycle, and high material utilization, and can manufacture any complex shape structure parts in theory [4,5,6]. Selective laser melting (SLM) is a technique, which utilizes a high-energy laser beam with a planned scanning strategy, used to scan pre-placed metal powders to make them melt rapidly. The powders are then cooled and solidified rapidly, and finally formed into solid components layer by layer [7,8].

SLM, one of the most important means of metal laser additive manufacturing, has attracted considerable attention in recent years, and its numerical simulation research has also become a hot issue in the industry. Due to the rapid movement of the laser heat source, the temperature of the material rapidly rises and then shows an extremely fast material solidification cooling rate. The temperature history of a locally fabricated area is extremely complicated, and the molten pool has a very short lifetime in a SLM process [9]. It is difficult to observe and record the transient temperature field, stress field, and molten pool morphology through experimental methods. Numerical simulation is an effective way to solve this problem.

To date, many scholars have carried out numerical simulation research on a metal powder SLM process. Chen et al. [10] established a multilayer finite element (FE) model to study the temperature field during a SLM process of TiB2/Ti6Al4V multi-material parts under various processing parameters. The simulation results showed that the maximum temperature gradient increased from 24.920 °C/μm to 37.754 °C/μm with increasing the laser power from 300 W to 450 W, but the maximum temperature gradient decreased from 33.884 °C/μm to 31.478 °C/μm with increasing the scanning speed from 400 mm/s to 1000 mm/s. Woo et al. [11] developed a three-dimensional FE model to investigate the thermal behavior during the SLM process of WC-reinforced H13 steel composite powder. The molten pool geometry under different parameters was analyzed based on the temperature field obtained by numerical simulation. The results showed that the distribution factor, packing efficiency, and absorption coefficient are the main factors affecting the molten pool structure. Ali et al. [12] developed a modeling approach to simulate the temperature field during a SLM process of Ti6Al4V powder. The validity of the model was validated by comparing the width and depth of the molten pool obtained by finite element analysis and experimental measurement. Based on this model, the effects of process parameters on cooling rates and temperature gradients inducing the accumulation of residual stress build-up was studied. Foroozmehr et al. [13] used a three-dimensional FE model to simulate the size of the molten pool in the SLM process. The model considered the penetration characteristics of laser beam on powder bed, and the depth depended on the thickness of powder. The temperature distribution, depth, width, and length of the molten pool of each track were studied, and the influence of scanning speed on them was analyzed. Wang et al. [14] developed a three-dimensional FE model to research the thermal behavior and residual stress during the SLM process of AlSi10Mg powder. The simulation results showed that the stress gradually increased during the SLM process due to the effect of heat accumulation, and the temperature gradient affected the final distribution of the residual stress. However, numerical studies have not yet been reported on the temperature field and stress field of 24CrNiMo alloy steel, an alloy steel material commonly used for processing brake discs for high-speed rail, fabricated by SLM, and the application of SLM technology in high-speed train and rail industry is rarely studied, which greatly restrict a wider application of this technology in the high-speed train and rail industry. Moreover, there are few researches on the influence of SLM scanning strategy on the temperature field and stress field.

In this paper, the distribution and evolution of temperature field and stress field of 24CrNiMo alloy steel by SLM are studied by finite element analysis method. The influence of different process parameters on temperature field distribution, temperature change rate, thermal stress evolution, and residual stress distribution is clarified. The influence rules and mechanisms of laser power, scanning speed, preheating temperature of substrate, subarea scanning strategy, and interlayer scanning angle changing on temperature field and stress field during a SLM process are revealed.

## 2. Modeling

### 2.1. Mathematical Model

**Figure 1:**
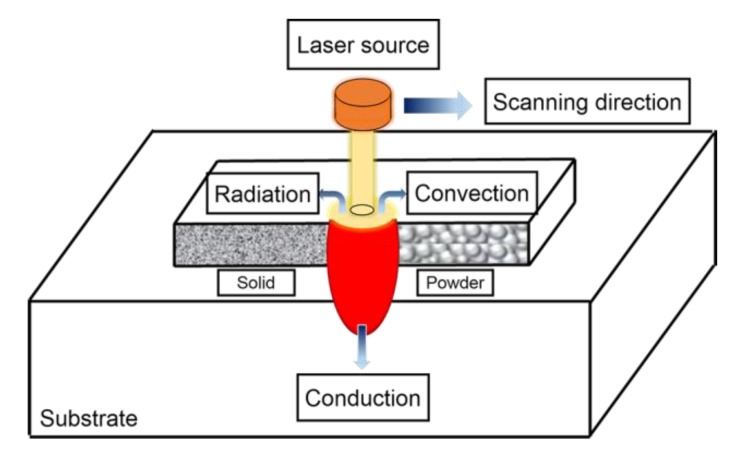
Schematic diagram of heat transfer during selective laser melting (SLM) process.

According to the basic theory of heat transfer, heat conduction, heat convection, and heat radiation are the basic modes of heat transfer. In a SLM process, metal powders melt rapidly and then solidify rapidly. The analysis of temperature field in this process is a non-linear transient thermal analysis, as shown in Figure 1.

For transient heat issues, based on Fourier heat transfer law and energy conservation law, and taking material anisotropic into account, the three-dimensional non-linear transient heat transfer control equation is

(1) $$\rho c\frac{\partial T}{\partial t}=\frac{\partial }{\partial x}(k_{x}\frac{\partial T}{\partial x})+\frac{\partial }{\partial y}(k_{y}\frac{\partial T}{\partial y})+\frac{\partial }{\partial z}(k_{z}\frac{\partial T}{\partial z})+Q,$$

where *k_x_*, *k_y_*, *k_z_* are the thermal conductivity in *x*, *y*, *z* direction, *T* is the powder temperature, Q is the heat flux density, *ρ* is the density, and *c* is the specific heat capacity.

Heat transfer between powder surface and ambient environment is heat convection and radiation. It can be described as follows:

(2) $$-k\frac{\partial T}{\partial n}=h(T_{a}-T_{s})+\sigma \varepsilon ({T_{a}}^{4}-{T_{s}}^{4}),$$

where *k* is the thermal conductivity, *n* is the normal vector to the powder surface, *T_a_* is the powder temperature, *T_s_* is the ambient temperature, *h* is the convection heat transfer coefficient, σ is the Stephen-Boltzmann constant (5.67 × 10^−8^ W/m^2^∙K^4^), and ε is the thermal emissivity.

### 2.2. Boundary Conditions

Considering the radiation impact of the laser on the powder, it is used as the correction coefficient of the boundary heat transfer. Moreover, the Equation (3) can be obtained by mathematical transformation of the Equation (2).

(3) $$-k\frac{\partial T}{\partial n}=\beta \left[h+\sigma \varepsilon ({T_{a}}^{3}+{T_{a}}^{2}T_{s}+T_{a}{T_{s}}^{2}+{T_{s}}^{3})\right]\times (T_{a}-T_{s}).$$

Total heat transfer coefficient is defined by:

(4) $$H=\beta \left[h+\sigma \varepsilon ({T_{a}}^{3}+{T_{a}}^{2}T_{s}+T_{a}{T_{s}}^{2}+{T_{s}}^{3})\right].$$

According to the experimental results of the temperature field from Reference [14], H at lower temperature is set to be thousands of times of that at steady state, and H at higher temperature is set to 80 times of that at steady state in this paper. Furthermore, at steady state, β=1.

The boundary condition is set to be three-point constraint [15], that is the three vertex constraints on the bottom of the substrate are $U_{x}=U_{y}=U_{z}=0$, $U_{y}=U_{z}=0$, $U_{z}=0$, respectively.

### 2.3. Heat Source Model

The double ellipsoidal heat source model used in this paper can be described as follows [16]:

(5) $$q(x,y,z)=\frac{6\sqrt{3}P\eta }{\pi \sqrt{\pi }abc}e^{\left(-3\frac{x^{2}}{a^{2}}-3\frac{y^{2}}{b^{2}}-3\frac{z^{2}}{c^{2}}\right)},$$

where *P* is the laser power, η is the laser absorptivity, *a*, *b*, and *c* are the shape parameters of the heat source, *x*, *y*, and *z* are the local coordinates.

### 2.4. FE Model

**Figure 2:**
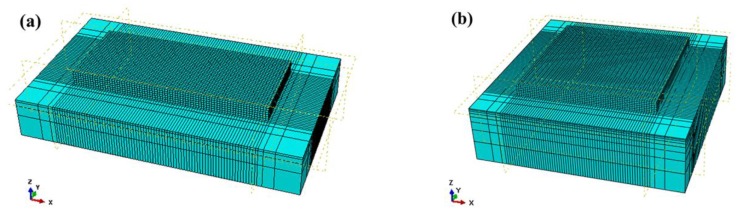
Finite element (FE) model: (**a**) model 1; (**b**) model 2.

In this paper, two models are used for analysis in ABAQUS (version 6.14.4, Dassault Systèmes, Paris, France), both of them include the substrate and the powder bed. As shown in Figure 2a, the dimensions of the substrate in FE model 1 are 2.0 mm × 1.2 mm × 0.3 mm, and the dimensions of the powder bed are 1.4 mm × 0.6 mm × 0.1 mm with two layers, the height of each layer is 0.05 mm. As shown in Figure 2b, the dimensions of the substrate in FE model 2 are 1.80 mm × 1.80 mm × 0.5 mm, and the dimensions of the powder bed are 1.2 mm × 1.2 mm × 0.10 mm with two layers, the height of each layer is 0.05 mm. Considering the computational efficiency and accuracy, the powder bed is meshed by DC3D8 element, which is an eight-node linear heat transfer brick element, with dimensions of 0.02 mm × 0.02 mm × 0.02 mm, and the substrate is gradually coarsely meshed away from the powder layer. The C3D8R element, an eight-node linear brick element, is used to calculate the stress field by applying the indirect coupling method based on the temperature field.

### 2.5. Material Properties

In this paper, the substrate material is 45 steel whose specific material properties are given in Reference [17]. The metal powder is 24CrNiMo alloy steel whose chemical composition is listed in Table 1.

**Figure 3:**
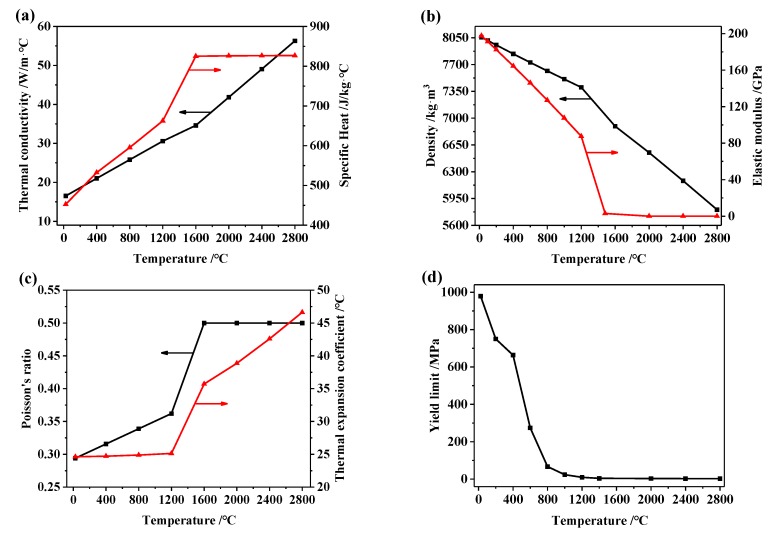
(**a**) Thermal conductivity and specific heat, (**b**) density and elastic modulus, (**c**) Poisson’s ratio and thermal expansion coefficient, and (**d**) yield limit of 24CrNiMo alloy steel.

The physical properties of 24CrNiMo alloy steel vary significantly with temperature, which should be considered in the simulation. In this paper, the temperature-dependent physical properties of 24CrNiMo alloy steel in solid state are obtained by the commercial software JMatPro (version 9.0, Sente Software Ltd., Surrey, UK), which is a powerful and integrated software for material properties simulation [18,19,20], as shown in Figure 3.

It is known that the temperature-dependent physical properties of 24CrNiMo alloy steel in powder state are quite different from those in solid state, including density, thermal conductivity, elastic modulus, and thermal expansion coefficient.

The effective thermal conductivity of the powder bed can be calculated by the Yagi-Kunii model [21]. It is described as follows:

(6) $$\frac{k_{e}}{k_{g}}=\frac{\beta \left(1-\varphi \right)}{\gamma \left(\frac{k_{g}}{k_{s}}\right)+\frac{1}{\frac{1}{\varphi }+\frac{D_{p}h_{rs}}{k_{g}}}}+\varepsilon \beta \frac{D_{p}h_{rv}}{k_{g}},$$

where φ is the porosity of the powder, *D_p_* is the average diameter of the powder particles, γ is the ratio of the solid effective length to the solid average diameter related to heat conduction, β is the ratio of the average length to average stacking diameter between two adjacent particles in the direction of heat flux, *k_e_*, *k_g_*, and *k_s_* are the effective thermal conductivity of the powder bed, the gas in the pores, and the solid material, *h_rs_* is the thermal emissivity between adjacent particle surfaces, and *h_rv_* is the thermal emissivity between adjacent gases.

The density of the powder bed can be calculated by Equation (7). It is described as follows:

(7) $$\rho_{p}=\left(1-\varphi \right)\rho_{s},$$

where *ρ_p_* is the density of the powder material, *ρ_s_* is the density of the solid material.

In the actual SLM process, Marangoni convection exists in the molten pool, resulting in the intensification of energy transfer, which has a significant effect on temperature and stress distribution. Therefore, the thermal conductivity above the melting point is modified by an enhancement factor to approximately consider the effects of Marangoni convection on heat transfer in the molten pool [22,23]. The modified thermal conductivity can be expressed as

(8) $$k^{*}(T)=\left\{ \begin{array}{ll} k(T) & T\leq T_{m}\\ ak(T) & T\geq T_{m} \end{array}\right.,$$

where *k(T)* is the actual thermal conductivity, k^*^(T) is the modified thermal conductivity, and *T_m_* is the melting temperature.

### 2.6. Latent Heat of Phase Transformation

In order to improve the calculation accuracy, the phase transformation latent heat of 24CrNiMo alloy steel during the SLM process is considered by applying the equivalent specific heat method. In the temperature range of phase transformation, the specific heat of the material can be modified by using the phase transformation latent heat [24]:

(9) $$C_{P}^{*}=C_{P}+\frac{L}{T_{L}-T_{s}},$$

where *L* is the phase transformation latent heat, *T_L_* is the liquidus temperature, *T_S_* is the solidus temperature, *C_P_* is the actual specific heat, and C_P_^*^ is the modified specific heat.

## 3. Results and Discussion

Firstly, it should be noted that the results of Section 3.1 and Section 3.2 are based on FE model 1 (Figure 2a) and the results of Section 3.3 is based on FE model 2 (Figure 2b).

### 3.1. Distribution and Variation Rules of Temperature Field and Stress Field

**Figure 4:**
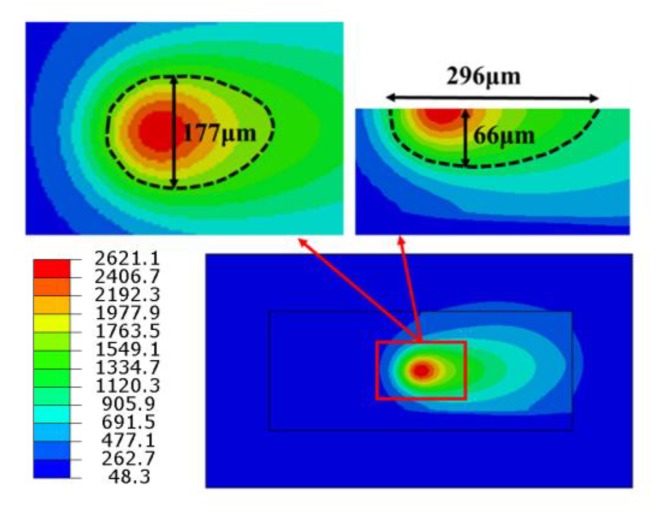
Temperature field distribution contour when the laser beam reaches the center of the first layer.

Figure 4 shows the temperature distribution contour of the whole model and the top surface and cross section of the molten pool under P = 250 W and v = 200 mm/s when the laser beam reaches the center of the first layer. It demonstrates that the peak temperature of the molten pool is 2621 °C and the isotherms on the front of the heat source are denser than those in the back, showing that the temperature gradient on the front of the heat source is larger. The main reason is that the front material of the heat source is still in powder state, and the heat cannot be transferred quickly because of its lower thermal conductivity, which results in a larger temperature gradient and a denser isotherm. The powder in the back of the heat source was transformed into solid, which increases the thermal conductivity and makes a faster heat transfer. The dotted line is the area where the temperature is higher than the melting point of the material (1435 °C), which can be used to characterize the structure of the molten pool. The length of the molten pool is about 296 μm, the width is about 177 μm, the depth is about 66 μm, the ratio of length to width is about 1.7, and the shape of the molten pool is similar to that of water droplets.

**Figure 5:**
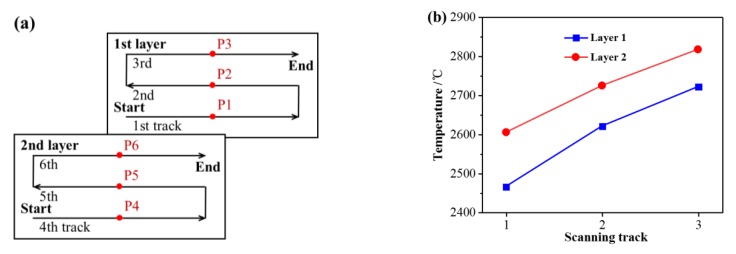
(**a**) Schematic diagram of the midpoint (P1–P6) on the top surface of each scanning tracks; (**b**) peak temperature at the midpoint of each scanning tracks.

Figure 5a shows the schematic diagram of the midpoint (P1–P6) on the top surface of each scanning tracks. The peak temperatures at the midpoint of the three tracks in the first layer are 2466 °C, 2621 °C, and 2722 °C, respectively, and those of the three tracks in the second layer are 2605 °C, 2725 °C, and 2818 °C, respectively, as shown in Figure 5b. As the laser scanning progresses the peak temperature increases with the increase of scanning tracks and scanning layers, but the increase amplitude decreases gradually.

**Figure 6:**
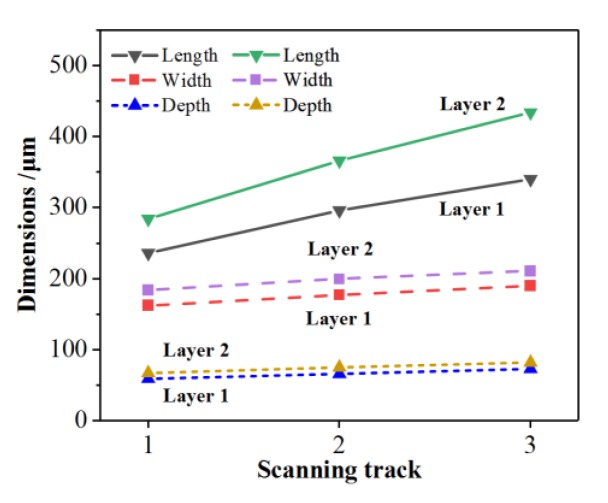
Three dimensions of the molten pool at the midpoint of each scanning tracks.

**Figure 7:**
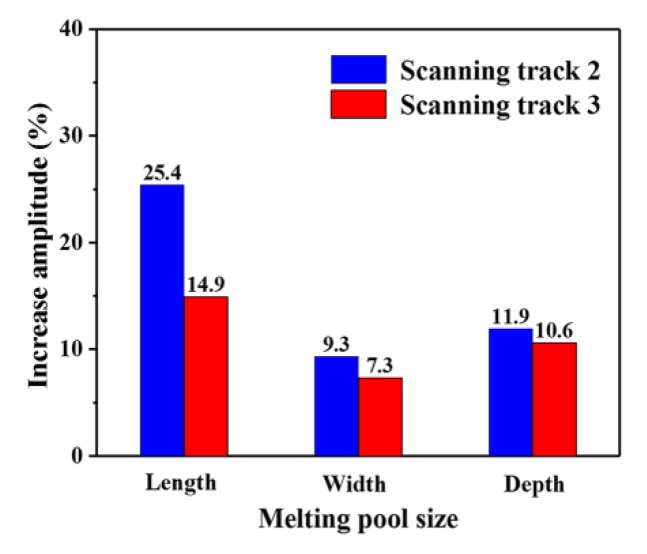
Increase amplitude of the molten pool size.

Figure 6 shows the three dimensions of the molten pool at the midpoint of each scanning track. The solid lines represent the length of the molten pool at the midpoint of each scanning track, the dashed lines represent the width of the molten pool at the midpoint of each scanning track, and the dotted lines represent the depth of the molten pool at the midpoint of each scanning track. As demonstrated, with the increase of scanning tracks, the length, width, and depth of the molten pool increase gradually. Furthermore, the three dimensions of the second layer molten pool are larger than those of the first layer to a certain extent. Taking the first layer as an example, the length, width, and depth of the molten pool at the midpoint of the first scanning track are 236 μm, 162 μm, and 59 μm, respectively. The length, width, and depth of the molten pool at the midpoint of the second scanning track are 25.4%, 9.3%, and 11.9% larger than those at the midpoint of the first scanning track, respectively. Moreover, the length, width, and depth of the molten pool at the midpoint of the third track are 14.9%, 7.3%, and 10.6% larger than those at the midpoint of the first scanning track, respectively. However, the increase amplitude of the three dimensions of the molten pool decreases gradually with the increase of the scanning tracks, as shown in Figure 7. Taking the second track of the two layers as an example, the length, width, and depth of the molten pool at the midpoint of the second track in the first layer are 23.6%, 13.0%, and 13.6% larger than those at the midpoint of the second track in the second layer, respectively. Combining with the temperature field, it is found that the three dimensions of the molten pool with different scanning tracks and layers increase in accordance with the variation of the peak temperature of the molten pool (see Figure 5), which indicates that the three dimensions of the molten pool are greatly affected by the temperature distribution of the molten pool. The reason for the decrease of the increase is that the heat transfer is slowed down with the increase of scanning tracks and layers.

**Figure 8:**
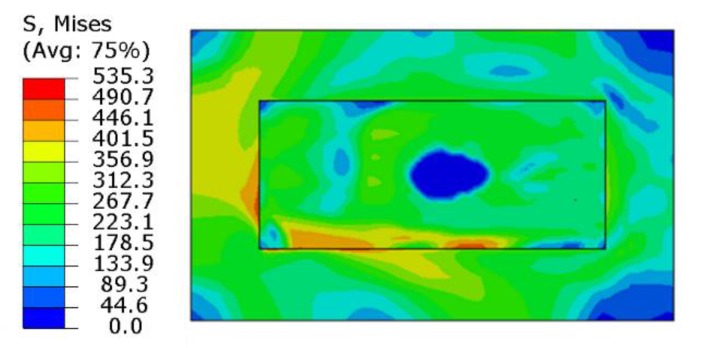
Stress field distribution contour when the laser beam reaches the center of the first layer.

When the laser beam scans the powder, the irradiated area of the spot absorbs a large amount of heat so that the temperature increases rapidly. After reaching the melting point, the powder state changes to a liquid state and then a molten pool is formed. At this time, the liquid phase is purely plastic, and the Mises stress is small and is approximately considered to be zero, as shown in Figure 8. When the laser beam is far away from this area, the temperature of the position decreases rapidly and the metal of the molten pool begins to solidify gradually. The material shrinks due to “thermal expansion and contraction”, and is bound by the solidified metal around it. The large temperature gradient results in the thermal stress increasing gradually. When the thermal stress is higher than the yield limit of the material, the solidified area is plastically deformed. As the molten pool moves continuously, the scanning area cools gradually. When the temperature tends to be stable, the thermal stress redistributes and remains in the SLMed parts.

**Figure 9:**
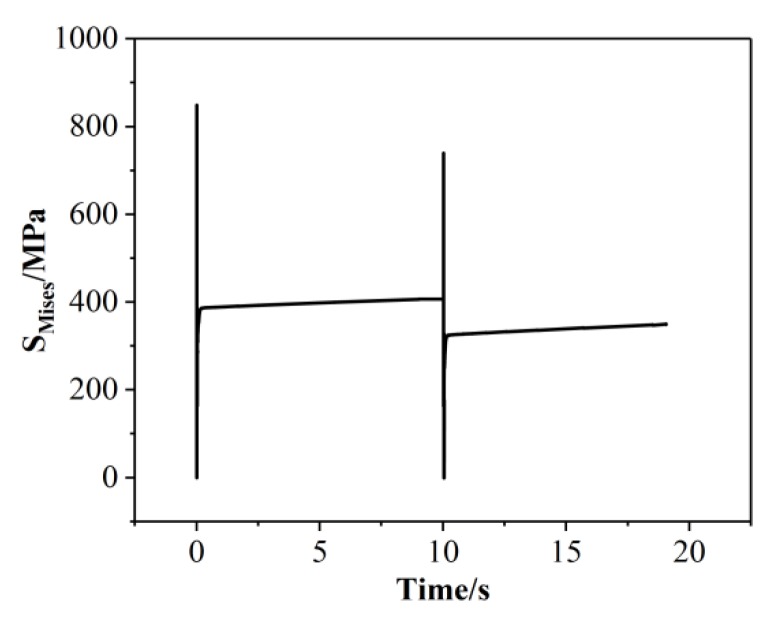
Evolution of stress at the center of the first layer.

Figure 9 shows the evolution of stress at the center of the first layer. When the laser beam scans the center of the first layer, the high temperature gradient results in a larger thermal stress before and after the molten pool, reaching more than 800 MPa. Moreover, the stress stabilizes at about 400 MPa with the cooling process at the end of the first layer. When the second layer is scanned, the position undergoes a similar temperature evolution with the first layer, but the temperature gradient decreases so that the peak stress decreases. Due to material shrinkage, the stress slightly increases during the cooling processing. The residual stress is released by a post-thermal effect and finally stabilizes at about 350 MPa.

**Figure 10:**
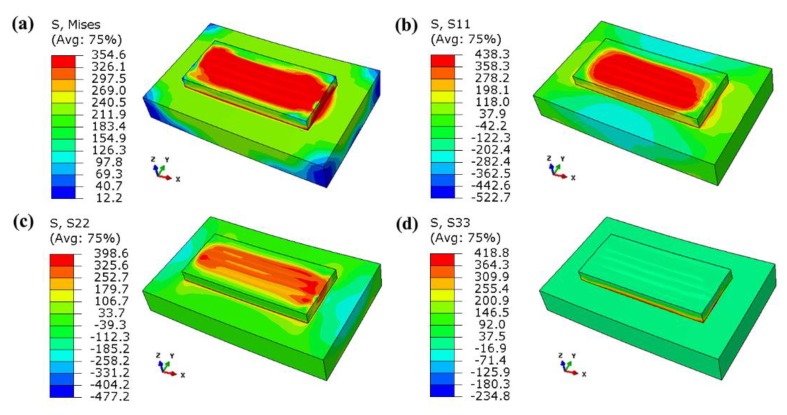
Residual stress distribution contour: (**a**) Von Mises stress, (**b**) X-direction stress, (**c**) Y-direction stress, and (**d**) Z-direction stress.

**Figure 11:**
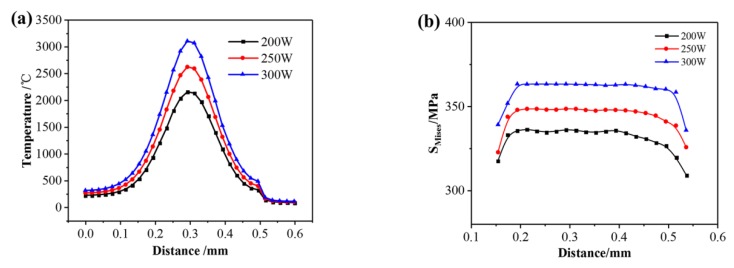
(**a**) Temperature distribution; (**b**) stress distribution under different laser powers.

Figure 10 shows the residual stress distribution contour after cooling. The maximum Mises stress is about 355 MPa, which appears at the joint edge of the substrate and the formed layer because of the large temperature gradient between them and the difference in thermal expansion coefficients of the materials. The high stress area is mainly concentrated in the formed layer, and the substrate stress is relatively low. It can be seen from Figure 11b,c that the residual tensile stress mainly occurs in the formed layer, and the X-direction residual stress is slightly higher than the Y-direction residual stress because the X-direction is the scanning direction, and the large cooling rate causes the stress to be parallel to the scanning direction. As can be seen from Figure 10d, the Z-direction residual stress is mainly concentrated between the substrate and the formed layer, which tends to cause warping and cracking.

### 3.2. Effects of Process Parameters on Temperature Field and Stress Field

Figure 11a shows the temperature distribution along the second scanning track under different laser powers when the laser beam scans the center of the first layer. The temperature distribution tends to follow the same rules, showing characteristics of high in the middle and low on both sides. The temperature and temperature gradient near the molten pool are relatively high in the middle area. When the laser power increases from 200 W to 300 W, the peak temperature of the molten pool increases accordingly, and the central temperature increases from 2153 °C to 3105 °C because the laser energy density and the heat input increases. Figure 11b shows the residual stress distribution of the second scanning track under different laser powers when scanning the center of the first layer. It can be seen that the Mises stress distribution rules are similar, and the stress in the middle of the scanning track is close to each other. As the laser power increases from 200 W to 300 W, the maximum of Mises stress increases from 335 MPa to 364 MPa. The main reason is that the increase of laser power has a great influence on the temperature gradient of the molten pool so that the growth rate increases from 6.9 × 10^3^ °C/mm to 1.2 × 10^4^ °C/mm during the cooling process.

**Figure 12:**
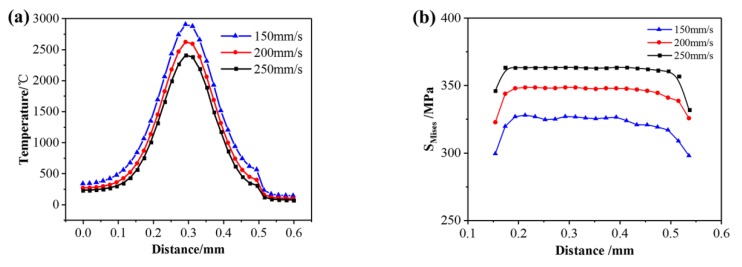
(**a**) Temperature distribution; (**b**) stress distribution under different scanning speeds.

Figure 12a shows the temperature distribution along the second scanning track under different scanning speeds when the laser beam scans the center of the first layer. When the scanning speed increases from 150 mm/s to 200 mm/s, the peak temperature of the molten pool decreases accordingly, and the central temperature decreases from 2905 °C to 2405 °C because the laser energy density and the heat input per unit time decreases. Figure 12b shows the residual stress distribution of the second scanning track under different laser speeds when the laser beam scans the center of the first layer. As the scanning speed increases from 150 mm/s to 200 mm/s, the maximum of Mises stress increases from 327 MPa to 363 MPa. The main reason is that the increase of scanning speed has a great influence on the temperature gradient of the molten pool so that the growth rate increases from 7.7 × 10^3^ °C/mm to 1.1 × 10^4^ °C/mm during the cooling process.

**Figure 13:**
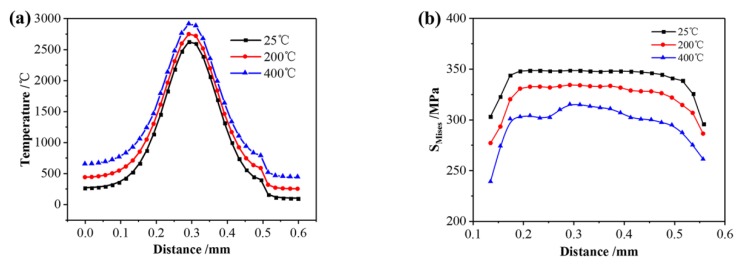
(**a**) Temperature distribution and (**b**) stress distribution under different preheating temperatures.

Figure 13a shows the temperature distribution along the second scanning track under different preheating temperatures when the laser beam scans the center of the first layer. When the preheating temperature increases from 25 °C to 400 °C, the peak temperature of the molten pool increases accordingly, and the central temperature increases from 2621 °C to 2914 °C because of the increase of heat accumulation. Figure 13b shows the residual stress distribution of the second scanning track under different preheating temperatures when the laser beam scans the center of the first layer. As the preheating temperature increases from 25 °C to 400 °C, the maximum of Mises stress decreases from 354 MPa to 300 MPa. The main reason is that preheating of the substrate helps to reduce temperature gradient. It also can be seen that the increase of the preheating temperature in a certain range makes the overall distribution of residual stress show a significant downward trend, and the high stress range of the formed area is gradually reduced, indicating that the preheating of the substrate helps to reduce the residual stress and the cracking tendency of SLMed parts.

### 3.3. Effects of Scanning Strategy on Temperature Field and Stress Field

**Figure 14:**
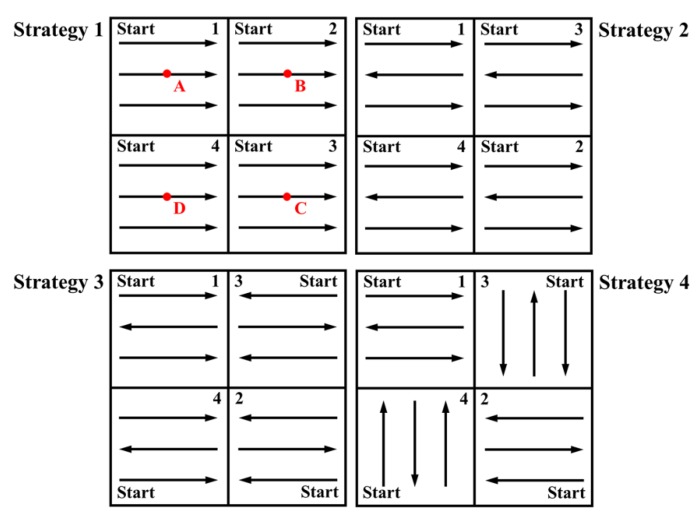
Schematic diagram of subarea scanning strategies.

Some differences exist in the temperature field under different subarea scanning strategies in the SLM process. The specific subarea scanning strategies are shown in Figure 14, and the scanning area is divided into four areas in which 1, 2, 3, and 4 represent the scanning sequence, “start” represents the starting point of scanning, and points A, B, C, and D are the area centers.

**Figure 15:**
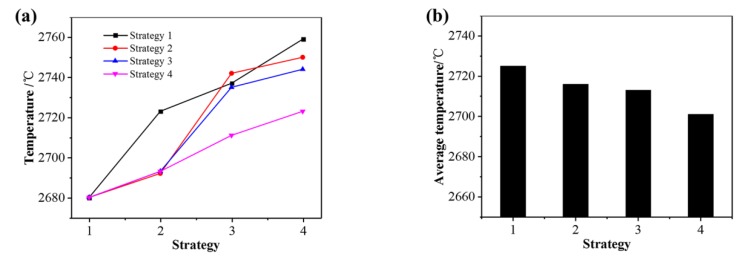
(**a**) Peak temperature of the area center and (**b**) average temperature under different subarea scanning strategies.

Figure 15a shows the peak temperatures of points A, B, C, and D under different subarea scanning strategies. The temperature of strategies 1, 2, 3, and 4 gradually increases due to the heat accumulation effect. Comparing Strategy 1 with Strategy 2, Strategy 2 has a lower temperature in Area 2 and 4, which indicates that the diagonal scanning can make the scanning area as far as possible to reduce the temperature increase caused by the thermal influence of the scanned area. Comparing Strategy 2 and Strategy 3, the temperature in Area 3 and 4 of Strategy 3 slightly decreases, indicating that the method of scanning from the edge to the center can also reduce the preheating effect. Comparing Strategy 3 and Strategy 4, Strategy 4 has a lower temperature in Area 3 and 4, indicating vertical scanning direction of adjacent areas that can reduce the influence of thermal accumulation from adjacent areas. In summary, far away from the scanning area, far away from the scanning starting point and adjacent areas with vertical scanning direction are the scanning strategies, which could result in a more uniform temperature distribution and a lower average temperature (as shown in Figure 15b), and help to reduce the residual stress caused by temperature difference.

**Figure 16:**
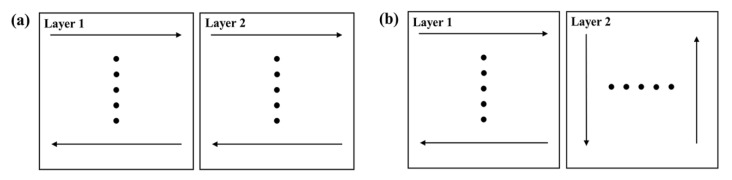
Schematic diagram of (**a**) no interlayer scanning angle and (**b**) 90° interlayer scanning angle.

**Figure 17:**
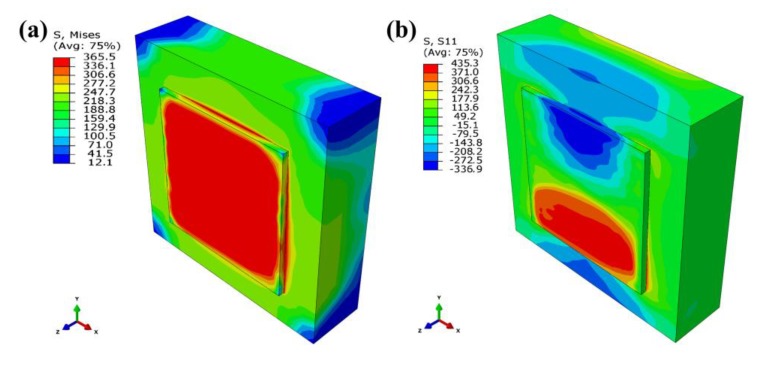

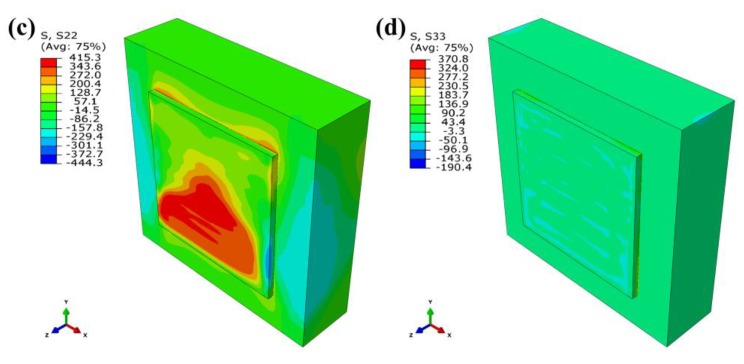
Residual stress distribution contour for scanning only the first layer: (**a**) Von Mises stress, (**b**) X-direction stress, (**c**) Y-direction stress, and (**d**) Z-direction stress.

Figure 16 shows the schematic diagram of the interlayer scanning angle changing. They have the same scanning starting point, the same scanning length of each tracks, and the same number of tracks. Figure 17 shows the residual stress distribution contour for scanning only the first layer after cooling. It demonstrates that the maximum Mises stress is about 366 MPa, and the stress distribution of the formed layer is relatively uniform, basically above 300 MPa. The substrate stress is lower, less than 200 MPa. The maximum tensile stresses in the X-direction, Y-direction, and Z-direction are 435 MPa, 415 MPa, and 371 MPa, respectively. The maximum compressive stresses in the X-direction, Y-direction, and Z-direction are 337 MPa, 444 MPa, and 190 MPa, respectively. Moreover, a large stress concentration occurs in the place where the formed layer is in contact with the substrate, which may cause cracking and warping.

**Figure 18:**
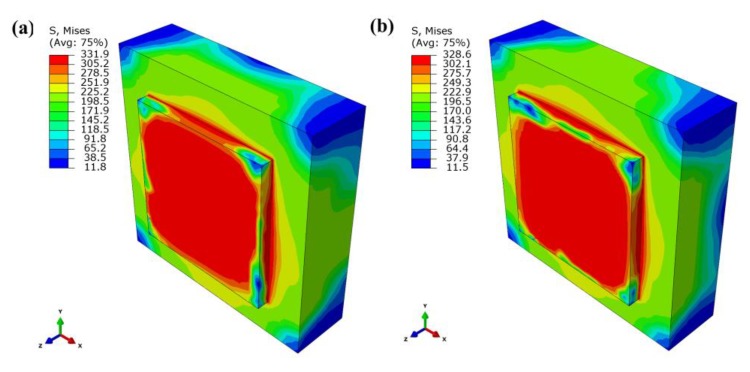

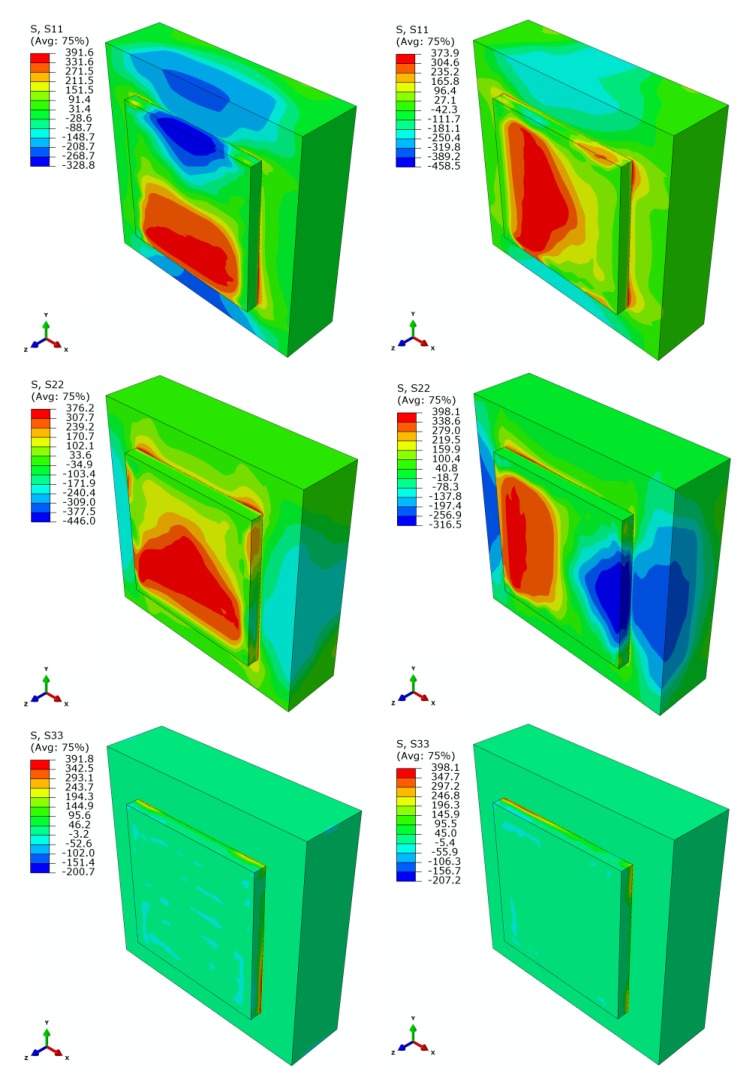
Residual stress distribution contour for scanning two layers with (**a**) no interlayer scanning angle and (**b**) 90° interlayer scanning angle.

Figure 18 shows the residual stress distribution contour for scanning two layers after cooling with no interlayer scanning angle and 90° interlayer scanning angle. It can be seen that the maximum Mises stress with no interlayer scanning angle is 332 MPa, and that of 90° interlayer scanning angle is 328 MPa. The maximum tensile stresses in the X-direction, Y-direction, and Z-direction with no interlayer scanning angle are 392 MPa, 376 MPa, and 392 MPa, respectively; the maximum compressive stresses in the X-direction, Y-direction, and Z-direction with no interlayer scanning angle are 329 MPa, 446 MPa, and 201 MPa, respectively; the maximum tensile stresses in the X-direction, Y-direction, and Z-direction with 90° interlayer scanning angle are 374 MPa, 398 MPa, and 398 MPa, respectively; the maximum compressive stresses in the X-direction, Y-direction, and Z-direction with 90° interlayer scanning angle are 459 MPa, 317 MPa, and 207 MPa, respectively. Meanwhile, by analyzing the residual stress values of the first layer, it is found that the stress with no interlayer scanning angle is lower than that with 90° interlayer scanning angle. The interlayer remelting under the 90° interlayer scanning angle causes the residual stress of the first layer to be released multiple times so that the stress of the first layer is reduced after redistribution. Therefore, the interlayer scanning angle changing helps to reduce the tendency of cracking by reducing the residual stress of the formed layer.

### 3.4. Experimental Verification

**Figure 19:**
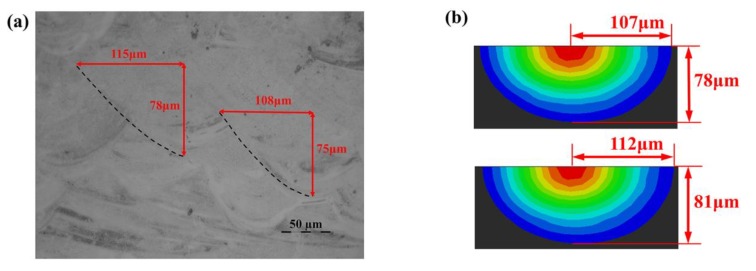
Comparisons of experimental (**a**) and numerical (**b**) morphology on the cross section of the molten pool.

Figure 19 shows the experimental and numerical molten pool morphology on the cross section of SLM processed specimens under P = 250 W and v = 200 mm/s. The average molten pool depth and half of the average molten pool width obtained by experiments are 76.5 μm and 111.5 μm, respectively, and those by numerical simulations are 79.5 μm and 109.5 μm, with errors of 5.8% and 2.9%, respectively (as shown in Table 2). It can be seen that the results of numerical simulation and experimental measurements are consistent with each other, which proves that the reliability and accuracy of the numerical analysis is good.

## 4. Conclusions

In this paper, the distribution and evolution of temperature field and stress field of 24CrNiMo alloy steel by SLM are studied by numerical simulation. The influence of process parameters (laser power, scanning speed, preheating temperature, and scanning strategy) on temperature field distribution, temperature change rate, thermal stress evolution, and residual stress distribution is analyzed, and the relevant experimental verification is carried out. The following conclusions are drawn:

(1) The peak temperature at the center of the first layer is 2621 °C, the length of the molten pool is about 296 μm, the width is about 177 μm, the depth is about 66 μm, the aspect ratio is about 1.7, and the shape is similar to that of water droplets. The increase of thermal accumulation leads to the increase of the peak temperature with the increase of scanning tracks and scanning layers, but the increase of that decreases gradually. The temperature field of the molten pool has great influence on the molten pool size. Temperature gradient results in large thermal stress around the molten pool, which reaches more than 800 MPa at the center of the first layer and stabilizes at about 400 MPa after the first layer is scanned. When the second layer is scanned, the temperature gradient decreases, and the residual stress is released post-thermally and finally stabilizes at about 350 MPa.

(2) When the laser power increases from 200 W to 300 W, the peak temperature of the molten pool increases from 2153 °C to 3105 °C, the maximum of temperature gradient increases from 6.9 × 10^3^ °C/mm to 1.2 × 10^4^ °C/mm during the cooling process, and the Mises stress increases from 335 MPa to 364 MPa. When the scanning speed increases from 150 mm/s to 200 mm/s, the peak temperature of the molten pool decreases from 2905 °C to 2405 °C, the maximum of temperature gradient increases from 7.7 × 10^3^ °C/mm to 1.1 × 10^4^ °C/mm during the cooling process, and the maximum of Mises stress increases from 327 MPa to 363 MPa. When the preheating temperature increases from 25 °C to 400 °C, the peak temperature of the molten pool increases from 2621 °C to 2914 °C, and the maximum of Mises stress decreases from 354 MPa to 300 MPa. Therefore, using appropriate process parameters help to reduce the residual stress.

(3) Far away from scanning area, far away from the scanning starting point and adjacent areas with vertical scanning direction are the scanning strategies, which could result in a more uniform temperature distribution, and help to reduce the residual stress caused by temperature difference. The interlayer remelting under the 90° interlayer scanning angle causes the residual stress of the first layer to be released multiple times so that the stress of the first layer is reduced after redistribution. Therefore, the interlayer scanning angle changing helps to reduce the tendency of cracking by reducing the residual stress.

## Figures and Tables

**Table 1:** Chemical composition of 24CrNiMo alloy steel powder.

Element	Fe	Cr	Ni	Mo	C	Mn	Al	P	S	Si	Nb
Content	Bal.	0.86	1.11	0.50	0.23	1.12	0.04	0.015	0.08	0.55	0.03

**Table 2:** Comparison of experimental and numerical molten pool dimensions.

Molten Pool Dimensions	Experimental Results (μm)	Numerical Results (μm)	Error
The average molten pool depth	76.5	79.5	3.9%
Half of the average molten pool width	111.5	109.5	1.8%
